# Risk factors for severe vasodilatory shock after cardiac surgery

**DOI:** 10.1186/cc14229

**Published:** 2015-03-16

**Authors:** J Almeida, F Galas, J Fukushima, E Almeida, A Gerent, E Osawa, C Park, R Nakamura, A Leme, M Sundin, R Kalil Filho, F Jatene, L Hajjar

**Affiliations:** 1 Heart Institute, São Paulo, Brazil

## Introduction

Vasodilatory shock is a well-known complication in patients who undergo cardiac surgery with cardiopulmonary bypass (CPB) and its occurrence is associated with higher morbidity and mortality. Despite that, clinical characteristics of vasoplegic shock and its spectrum of severity are poorly described. The aim of this study was to compare patients who developed mild to moderate vasoplegic shock with patients who developed a severe form and to identify predictive factors for the severe form of vasoplegic shock.

## Methods

We performed an observational study in 300 patients who underwent cardiac surgery with CPB and presented within the first 24 hours after surgery with refractory hypotension and used a vasopressor agent. Severe vasoplegic shock was defined as a requirement of norepinephrine higher than 1 μg/kg/minute or the use of two or more vasopressors. Baseline characteristics, laboratorial, clinical and intraoperative data, such as amount of fluids, bleeding, blood transfusion, inotropes and length of CPB were collected at ICU admission. Logistic regression was performed using severe vasodilatory shock as the outcome.

## Results

There were 46 (15%) patients who develop the severe form of vasodilatory shock within 24 hours after cardiac surgery. In a univariate analysis, patients with the severe form were more likely to be older, to receive more blood transfusion and inotropic agents, to have higher levels of serum lactate, lower hemoglobin concentration and lower SvO_2_ at the end of the procedure, lower cardiac output index, higher heart rate and higher levels of reactive C protein at ICU admission. These patients also experienced more postoperative organ dysfunction, had a longer length of ICU stay and higher mortality. There were no differences between patients regarding amount of fluids and length of CPB. In a multivariate analysis we identify age (OR = 1.04, 95% CI = 1.01 to 1.08, *P *= 0.016), intraoperative use of epinephrine (OR = 5.49, 95% CI = 2.42 to 12.43, *P *< 0.001), higher serum lactate at the end of the procedure (OR = 1.04, 95% CI = 1.01 to 1.06, *P *= 0.001) and intraoperative blood transfusion (OR = 5.06, 95% CI = 2.19 to 11.69, *P *< 0.001).

## Conclusion

This study demonstrated that older patients, intraoperative blood transfusion and utilization of epinephrine were independently associated with a more severe form of vasodilatory shock after cardiac surgery with CPB. Also, we identified that a higher lactate at the end of the procedure was an independent predictive factor for this severe form of shock.
